# Is increased activation in the fusiform face area to Greebles a result of appropriate expertise training or caused by Greebles' face likeness?

**DOI:** 10.3389/fnins.2023.1224721

**Published:** 2023-10-17

**Authors:** Kuo Liu, Chiu-Yueh Chen, Le-Si Wang, Hanshin Jo, Chun-Chia Kung

**Affiliations:** ^1^School of Psychological and Cognitive Sciences and Beijing Key Laboratory of Behavior and Mental Health, Peking University, Beijing, China; ^2^Department of Psychology, National Cheng Kung University, Tainan, Taiwan; ^3^Brain & Cognition, Leuven Brain Institute, KU Leuven, Leuven, Belgium; ^4^Institute of Creative Industries Design, National Cheng Kung University, Tainan, Taiwan; ^5^Institute of Medical Informatics, National Cheng Kung University, Tainan, Taiwan; ^6^Mind Research and Imaging (MRI) Center, National Cheng Kung University, Tainan, Taiwan

**Keywords:** fusiform face area (FFA), Greeble training, ROI analysis, adaptation effect, neural inversion effect (NIE), multi-voxel pattern analysis (MVPA)

## Abstract

**Background:**

In 2011, Brants et al. trained eight individuals to become Greeble experts and found neuronal inversion effects [NIEs; i.e., higher fusiform face area (FFA) activity for upright, rather than inverted Greebles]. These effects were also found for faces, both before and after training. By claiming to have replicated the seminal Greeble training study by Gauthier and colleagues in 1999, Brants et al. interpreted these results as participants viewing Greebles as faces throughout training, contrary to the original argument of subjects becoming Greeble experts only *after* training. However, Brants et al.'s claim presents two issues. First, their behavioral training results did not replicate those of Gauthier and Tarr conducted in 1997 and 1998, raising concerns of whether the right training regime had been adopted. Second, both a literature review and meta-analysis of NIEs in the FFA suggest its impotency as an index of the face(-like) processing.

**Objectives:**

To empirically evaluate these issues, the present study compared two documented training paradigms Gauthier and colleagues in 1997 and 1998, and compared their impact on the brain.

**Methods:**

Sixteen NCKU undergraduate and graduate students (nine girls) were recruited. Sixty Greeble exemplars were categorized by two genders, five families, and six individual levels. The participants were randomly divided into two groups (one for Greeble classification at all three levels and the other for gender- and individual-level training). Several fMRI tasks were administered at various time points, specifically, before training (1st), during training (2nd), and typically no <24 h after reaching expertise criterion (3rd).

**Results:**

The ROI analysis results showed significant increases in the FFA for Greebles, and a clear neural “adaptation,” both *only* in the Gauthier97 group and *only after* training, reflecting clear modulation of extensive experiences following an “appropriate” training regime. In both groups, no clear NIEs for faces nor Greebles were found, which was also in line with the review of extant studies bearing this comparison.

**Conclusion:**

Collectively, these results invalidate the assumptions behind Brants et al.'s findings.

## Introduction

Face recognition is an enduring and intriguing research topic in psychology and visual neuroscience. This is partly because of its importance to our social lives and species survival (Rivolta, 2014) and partly because it showcases the intricate interplay between “nature” and “nurture” (Sugita, 2008; Arcaro et al., 2017). Since the first uses of PET and fMRI in research, studies in face recognition have identified several related processing regions (Sergent et al., 1992; Haxby et al., 1996). However, it was not until 1997, when the term fusiform face area (FFA) was coined (Kanwisher et al., 1997), that investigation of this area began in earnest. Since then, at least two opposing views (i.e., face specificity and perceptual expertise) have surfaced and continue to be discussed to this day (Gauthier and Tarr, 1997; Kanwisher, 2000, 2017; Tarr and Gauthier, 2000). As claimed by supporters of the domain-specific hypothesis, neural processing in the FFA is specific to faces (or face-like objects), less recruited by other objects, and more likely to be inherited by genetic endowment (Grimaldi et al., 2016). Conversely, proponents of the perceptual expertise hypothesis assert that experience plays a vital role in face-processing mechanisms. Faces are something most humans see every day from birth, making it incredibly likely that life-long experience with faces underpins our species' superb recognition capability. If this is true, the FFA should not only be activated in response to faces but also in response to “expert” object categories, such as car experts viewing cars or radiologists viewing X-ray images (Gauthier et al., 2000a).

To test and verify the expertise hypothesis, Gauthier and Tarr (1997) created Greebles—an artificial object set, for which they trained participants to recognize at various levels (i.e., sex, family, and individual). Participants were familiarized with various Greebles and fMRI-scanned before and after 8–10 training sessions. In one seminal fMRI study (Gauthier et al., 1999), three lines of evidence jointly supported the expertise hypothesis: specifically, (a) the decreased response times (RTs) for verification tasks with Greebles, to the point of statistical insignificance between RTs for identifying Greebles at the family level and those at the individual level; (b) the “surfacing” of the FFA by the “Faces vs. Objects” contrast before training, and by both “Faces vs. Objects” and the “Greebles vs. Objects” contrasts after training, suggesting that training “drove” Greeble selectivity in the FFA; and (c) comparison of activation levels before and after training found a neural inversion effect (NIE) closely associated with training (i.e., the sum of *t*-values in the right FFA for upright vs. inverted faces decreased, while for Greebles concurrently increased, and the gap of summed *t*-values between faces and Greebles significantly interacted). Along with studies of natural experts (Gauthier et al., 2000a; Xu, 2005), the perceptual expertise hypothesis has received decent support (Bukach et al., 2006). However, upon follow-up, some heated exchanges ensued (Grill-Spector et al., 2004; McKone and Kanwisher, 2005; Gauthier and Bukach, 2007; McKone et al., 2007; Op de Beeck and Baker, 2010a,b).

Among objections to the expertise hypothesis of the FFA, the most relevant was a study by Brants et al. (2011), who claimed that the FFA's increased response to Greebles was due to their resemblance to faces. Brants et al. claimed that, by replicating Gauthier's classic training paradigm (Gauthier and Tarr, 1997), they found NIEs in the FFA, as assessed by significantly higher blood-oxygen-level dependent (BOLD) activity for upright than for inverted stimuli (either faces or Greebles), both before and after training. In addition, Brants et al. found that either encouraging or discouraging subjects from seeing Greebles as “living individuals” or “objects” did not matter; all subjects reported perceiving Greebles as “face-like” after training. Based on the post-training interview and the NIE observed for faces and Greebles, both before and after training, Brants et al. concluded that it was Greebles' perceived face likeness, rather than acquired expertise, that drove the NIE, the presumed indicator of face selectivity for the FFA.

Upon closer inspection, however, four inconsistencies (two behavioral and two fMRI) between Gauthier et al. (1999) and Brants et al. (2011) could be identified. First, as the basic premise of a successful replication is an almost identical, or at least comparable, behavioral result (typically a prerequisite for the later fMRI findings), Brants et al. (2011) reported mean RTs for accurate verification trials of ~1,000 ms. This was nearly double the RTs (~500 ms) reported in the original study (figure 3 of Gauthier and Tarr, 1997, p. 1677). Although Brants et al. (2011), p. 3951, stated that “All aspects of the procedure were modeled after Gauthier and Tarr (1997), except the manipulation of inducing face likeness for half of the participants,” they nevertheless also stated “ten Greebles remained unknown throughout the training” (p. 3951), which happened neither in the study of Gauthier and Tarr (1997) nor in their study in 1999 (Gauthier et al., 1999). Such a “half-trained, half-untrained” arrangement of Greebles more resembles the training procedure of Gauthier et al. (1998), where the verification RTs for the two trained categories (e.g., sex and individual) differed significantly (ibid, figure 4B, p. 2407), and the mean verification RT of ~1,050 ms (averaging the former mean of ~800 ms and the later mean of ~1,300 ms) was also more consistent with those reported by Brants et al. (2011, figure 3b, p. 3953). These observations and reasoning suggest that Brants et al. (2011) adopted a different or variant of the training paradigm (i.e., Gauthier et al., 1998) than those originally used in Gauthier and Tarr (1997) and Gauthier et al. (1999). However, it would still be necessary to empirically compare the effects of different expertise training in the FFA/inferior occipitotemporal cortex to draw more precise conclusions. Second, Brants et al. (2011) used the “statistical equivalence between mean verification RTs between family and individual level” (Gauthier and Tarr, 2002) as the sole criterion for subjects to reach perceptual expertise. However, according to Tanaka and Gauthier (1997), two other implicit criteria also need to be met to jointly define “perceptual expertise,” which are as follows: (a) that the RTs between Greeble verifications at the family and those at the individual levels are supposed to be large and/or significantly different, with the latter usually longer; and (b) the RT differences would monotonically decrease to the point of near convergence or statistical insignificance. With only one criterion (i.e., statistical insignificance, as depicted by three arrows; sessions 1, 2, and 8, respectively; see Brants et al. (2011, figure 3b, p. 3953), it is unimaginable that novices could behave like experts at the beginning and at the end of, but not in between, the training sessions.

Third, while Gauthier et al. (1999) and Brants et al. (2011) reported behavioral training results and comparisons of inversion effects in the FFA for faces and Greebles, the former provided additional contrast images of category-selective regions. As shown in Gauthier et al. (1999, figure 4, p. 571), before training, the FFA could only be identified in the “Face vs. Object” contrast. After training, however, contrasts between “Face vs. Object” and “Greeble vs. Object” revealed the same FFA. If, according to Brants et al. (2011), participants interpreted Greebles as faces throughout training, further convincing support would be to similarly show FFA activations for “Face/Greeble vs. Object” contrast before *and* after training. While these were not seen in Brants et al. (2011), a counterargument to the “Greebles look like faces” claim could be that since most of us do sometimes consider Greebles “face-like” *before* (or without any) training, the fact that “Greeble vs. Object” contrast revealed FFA only *after*, but not *before*, training, as revealed in Gauthier et al. (1999), strongly suggest the necessity of sufficient training to shift processing from the basic to subordinate level (Gauthier et al., 1997, 2000b) as the crucial factor driving increased FFA activity for Greebles, not by Greebles' face-resemblance alone. Fourth, as both Gauthier et al. (1999) and Brants et al. (2011) observed NIEs in the FFA as the indispensable evidence for their respective claim, the reliability of NIE in the FFA as an index of face processing was, a bit surprisingly, hardly examined. Furthermore, the above two studies adopted different dependent measures: summed *t*-values (Gauthier et al., 1999) and average percent signal changes, or PSC (Brants et al., 2011), across FFA voxels. As behavioral inversion effects may not be face-specific (Valentine, 1988; Rossion and Gauthier, 2002), our extant literature search by keywords such as “FFA” and “face inversion” rendered 18 published articles (shown in Table 1). Not only did these articles show surprisingly inconsistent NIE effects at FFA but further funnel plots also suggested that the NIE in the FFA was never unanimously one-sided (larger for upright orientation). In light of the apparent unreliability of NIEs as an indicator of face-specific processing, it may be worthwhile to seek an additional neuronal measure of face/expertise processing.

**Table 1:** Summary literature of the neural inversion effect of the face in FFA.

**References**	**fMRI design**	**Number of subjects**	
Kanwisher et al. (1998)	Blocked	10	*F*_(1, 9)_ = 10.6, ^*^p < 0.01
Yovel and Kanwisher (2004)	Blocked	17	*F*_(1, 13)_ = 16.5, ^*^p < 0.001
Yovel and Kanwisher (2005)	blocked + er_fMRI	21 + 14	*t*_(30)_ = 4.67, ^*^p < 0.001
Passarotti et al. (2007)	Blocked	3 groups, *N* = 30	Pos. NIE for adults [*F*_(1, 27)_ = 4.81, ^*^p < 0.04]
Rhodes et al. (2009)	Blocked	16	*F*_(3, 39)_ = 17.34, ^*^p < 0.0001, eta-square = 0.57
Brants et al. (2011)	Blocked	8	*F*_(1, 7)_ = 12.083, ^*^p < 0.05
James et al. (2013)	Blocked	12	*t*_(11)_ = 4.92, *q* < 0.05 (Figure 4)
Haxby et al. (1999)	Blocked	6	Not sig.
Aguirre et al. (1999)	Slow event-related	8	*t*_(7)_ = 0.88, not sig.
Epstein et al. (2006)	Blocked	12	*p* > 0.15
Kung et al. (2007)	Blocked	10	Not sig. [*t*_(18)_ = 0.662, *p* = 0.26]
Strother et al. (2011)	Blocked	12	not sig. (judged by Figures 4A, 5)
Bilalić et al. (2011)	Blocked	15 (7 experts and 8 novices. in Exp1)	Not sig. (stats unreported. Figure 1D)
Grotheer and Kovács (2014)	Blocked	26	*F*_(1, 25)_ =0.09, (^*^p < 0.05)
Matsuyoshi et al. (2015)	Event-related	32	Not sig. (Figure 5)
Chou et al. (2021)	Event-related	32	Not sig (Figure 3B)
The present study	Blocked	16	Only 1 out of 6 was sig (Figure 8)
Bookheimer et al. (2008)	Blocked	12 (ASD) and 12 ND	Reversed NIE *F*_(1, 154)_ = 38.73, *p* < 0.001
Passarotti et al. (2007)	Blocked	3 groups, *N* = 30	Reversed NIE for children [*F*_(1, 10)_ = 11.86, *p* < 0.002]

Of the 18 articles, seven of them found a positive (i.e., larger activity for upright than for inverted faces) significant NIE (Green cell points), nine of them did not find significant NIE (Yellow cell points, with five of them in darker yellow colors, indicating their lack of statistical, t/p/F, values), and two found a reverse NIE (Blue cell points). * means significant NIE reported in literature.

With these four comments, Gauthier and Tarr (1997), Gauthier et al. (1998, 1999), and Brants et al. (2011), the purpose of the present study was to revisit the two Greeble training regimes and to compare their differential interactions with the FFA. To show that FFA selectivity is not driven by Greebles' face likeness (but more likely by its subordinate-level processing), we chose asymmetric Greebles to minimize the effect of symmetry. If all putative Greeble selectivity in the FFA could still be observed for the asymmetric version, stimulus symmetry (i.e., a potential factor behind face likeness) becomes less important in shaping the selectivity of the FFA. Additionally, in the current experiment, both the FFA and lateral object complex (LOC) would be independently localized (c.f., Brants et al., 2011). Other experimental procedures, including the Greebles-Objects-Faces (GFO) tasks and the face/Greeble inversions manipulation, were kept as closely matched with those in both Gauthier et al. (1999) and Brants et al. (2011).

The domain-specific hypothesis predicts that Greeble training does not affect FFA but could be on another object-selective area (e.g., LOC). In contrast, the perceptual expertise hypothesis predicts that the “Greeble vs. Object” contrast should reveal the FFA *only after* training. We predicted that different training regimes would also yield different response profiles and different Greeble selectivities in the FFA accordingly. Regarding NIEs at the FFA, we are equivocal about the direction of NIE in the FFAs since the funnel plot suggests that positive, negative, or no different NIEs are all possible. Finally, because the expertise hypothesis predicts that if the FFA becomes similarly responsive to faces and Greebles after appropriate training, we would expect neural adaptation (Grill-Spector et al., 1998; Grill-Spector and Malach, 2001) or competition in the FFA: for example, reduced FFA activities for faces right after Greebles, but only after training, and only under the right training regime.

## Materials and methods

### Behavioral experiment

#### Participants

To justify the choice of subject number with the appropriate statistical power, we ran G^*^Power with the estimated RT data: one-tailed between-group *t*-test, with effect size = 2 (RT = 2:1 in pre- vs. post-training), alpha = 0.05, power = 0.96, resulting in *N* = 7 in each group (*N* = 14), enough to show a reliable difference. We, therefore, recruited 16 NCKU undergraduate and graduate students (nine girls), all with normal or corrected-to-normal vision. After signing the informed consent approved by the NCKU Research Ethics Committee (REC case number 103-266), participants were randomly assigned into two separate training paradigms (*n* = 8 in each), which included three fMRI scans (before, during/halfway, and after training) and 10 sessions of behavioral training in between, lasting ~2 weeks. At the end of the training, all participants received a cash payment of ~NT$ 6,000 (or ~US$ 200).

#### Materials

**Figure 1:**
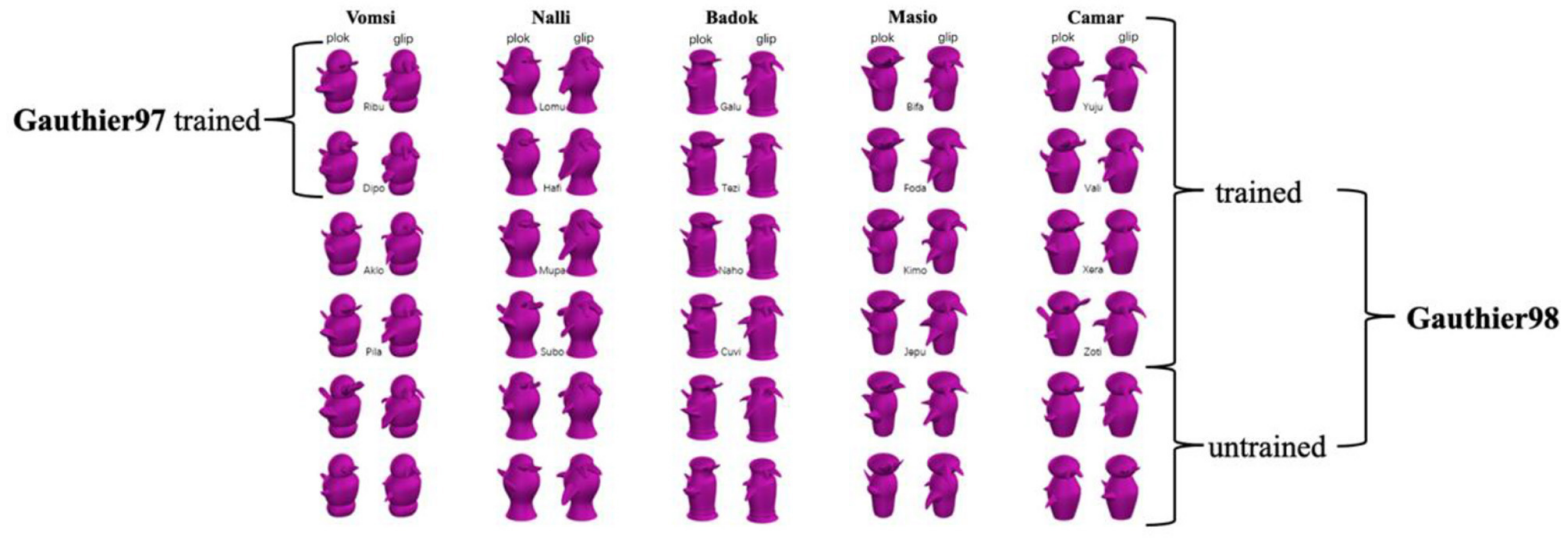
Thirty asymmetric Greebles were used in the present study, with each column representing a family (a total of 5 with family names above); each family contains six individuals each (names in between the two trained views), which are presented equally throughout the training process. There are three differences between Gauthier97 and Gauthier98: First, Gauthier97 has a family level to learn, whereas Gauthier did not; Second, Gauthier97 had 10 individual names to learn, while Gauthier98 had 20. Third, Gauthier98 added an unnamed label in the naming and verification task. All Greeble stimuli were downloaded from https://sites.google.com/andrew.cmu.edu/tarrlab/stimuli.

The 60 Greeble exemplars (Figure 1) are categorized by two genders, five families, and six individual levels (also see Gauthier and Tarr, 1997, figure 1, p. 1674). Gender is defined by Greebles' upward or downward “appendages” (i.e., protrusions on the top and main body), the family by the five different body shapes, and the individual by the unique combination of body shape and the appendages. In addition, Figure 1 shows the meaningless syllables assigned to these asymmetric Greebles as their respective gender, family, and individual name, with all the initial letters different for the convenience of responses in the later naming task.

Both training regimes were performed in MATLAB 2010a on an iMac with a resolution of 1,024^*^768. All Greeble models were presented in purple shade (Figure 1). The size of the presented images was ~6.5 cm high and 3.25 cm wide (nearly identical to Brants et al., 2011). Every participant saw Greebles in the center of the screen, which was placed at a distance of 60 cm, subtending an ~6.2° × 3.1° visual angle.

#### Procedure

The 16 participants were randomly divided into two groups (i.e., training regimes). The first group followed the training procedure of Gauthier and Tarr (1997) and Gauthier et al. (1999) (shortened to Gauthier97 hereafter), and the other followed those by Gauthier et al. (1998) and Brants et al. (2011) (shortened to Gauthier98 hereafter). As stated in Gauthier et al. (1998, p. 2403), there were three explicit procedural differences between the Gauthier97 and Gauthier98 groups. First, Gauthier97 participants were trained to classify Greebles at all three levels (gender, family, and individual), whereas Gauthier98 only included gender- and individual-level training. Second, Gauthier97 trained participants to discriminate 10 different Greebles (two from each gender and five from each family), as opposed to 20 in the Gauthier98 protocol. Finally, Gauthier97 had participants to perform only the verification task during training, while Gauthier98 required participants to alternate between the verification and naming tasks during most training sessions. Another outstanding but less clearly stated difference was the treatment of unnamed Greebles during training (also see Figure 1 of the present study). Of the 60 Greebles (or 30, if picking only one gender per family), only the chosen 10 were trained at the individual level in the Gauthier97 protocol, leaving the remaining 20 Greebles for the training at the gender and family level, respectively (10 for each). In contrast, in the Gauthier98 protocol, other than the 20 trained Greebles, the remaining 10 unnamed ones were mixed with 20 trained ones during training at both gender and individual levels, creating additional responses in both naming (pressing the letter “u” for “unnamed”) and verification (pressing spacebar for “null” responses) tasks. We believe, as we will also explain later in **Figure 11**, that these are the primary reasons driving the RT differences in the verification task between Gauthier98 and Gauthier97. Except for these differences, all other aspects of the training regimes, including the trial structure (e.g., time intervals, stimuli addition order (two per day for the first five training days, then level off the second 5 days), and a “beep warning sound after incorrect trials”), mimicked as closely as described in both Gauthiers' protocols. All 16 participants (eight in each training regime) completed 10 training sessions (~2 weeks), each lasting ~40 min. At the end of each naming/verification task block, mean accuracy and mean RT of accurate trials were shown on the screen. Finally, at the beginning and end of the training, we also asked each participant questions like “What do you think the Greebles look like?” and “Were the Greebles ever face-like to you at any time during training?”

For data analysis, accuracy and RTs for correct responses across each training group of participants (*n* = 8) were averaged. Throughout the study, we used the same definition of expertise criterion (Kanwisher et al., 1997; Gauthier et al., 2000a): namely, the statistical insignificance between correct RTs at the individual vs. the family (Gauthier97)/gender (Gauthier98) level in the verification task.

### fMRI

Several fMRI tasks were administered at various time points: specifically, before training (1st), during training (2nd), and typically no <24 h after reaching the expertise criterion (3rd).

#### Greeble-Face-Object task

Present in both Gauthier et al. (1999) and Brants et al. (2011), the GFO task is necessary for both examination of training effects and cross-study comparisons. It requires subjects to passively view two runs (322 s; 161 volumes each) of 12 image blocks (each category repeated four times, 20 images per block, and stimulus-onset asynchrony of 1 s), sandwiched between 6-s fixation periods (first fixation lasting 10 s for dummy scan), in both before- and after-training fMRIs. Unique in the current protocol, to assess the relative interference or neural adaptation (e.g., face stimuli presented after the Greeble block), each stimulus category was with equal probability before and after the other two categories, so that the four face blocks would be twice after Greebles and twice after objects, with the same being true for both Greebles and objects.

#### NIE task

**Figure 2:**
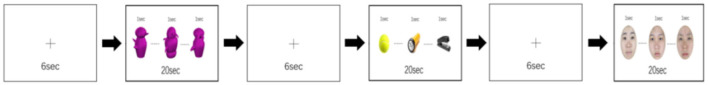
An example of GFO (Greeble, Face, Object) task used in fMRI scan. Stimuli were randomly presented for 1 s each during a 20 s block, while fixation blocks (6 s) in between each stimulus block. Gauthier used this for localizer but here different localizer task was used.

Also adopted from both Gauthier et al. (1999) and Brants et al. (2011, see Figure 2, p. 3952 for illustration), each NIE run contained four blocks of sequential matching trials (eight per block), where each trial consisted of fixation (800 ms), followed by consecutive presentations of stimulus (1 s for each image, separated by a 200 s mask in between). Four categories (Greebles/faces) by orientation (upright/inverted) combinations were block-randomized in each run, and fMRI data from five runs were collected both before and after the training session. Behavioral reaction time and accuracy inside the scanner were also recorded.

#### FFA localizer task

To independently assess the differential effects of training on GFO activities, we additionally ran an independent FFA localizer (from Peelen and Downing, 2005) for each participant. Participants performed a one-back identity judgment for faces, bodies, scrambled objects, and Greebles (arranged in +ABCD+, a pseudo-random fashion) for two runs. In addition to FFA, an object-selective region (i.e., the LOC) was circumscribed for companion analyses.

#### Scan parameters and analysis

The fMRI data were acquired from a GE750 3T scanner housed in the NCKU MRI Center, with a 32-channel head coil. Parameters were set to TR = 2,000 ms, TE = 33 ms, 64 × 64 matrix (so that FOV = 19.2 × 19.2 cm), 3 mm slice thickness (without gaps, making the voxel size = 3 × 3 × 3 mm^3^), and 40 axial slices, covering the whole brain.

Data were analyzed by BrainVoyager QX (BVQX v2.6) and NeuroElf 1.0 (http://neuroelf.net) under MATLAB 2018a. Functional data preprocessing included slice-time correction and motion correction. The resulting functional (T2^*^) images were coregistered (with the normalized T1 image in the Talairach space) and then transformed into 3D volume time course (VTC) data, which was then entered into a generalized linear model (GLM) and contrast analysis. Cross-session alignments of the anatomical images were performed for the first session (before training) so that all subsequent volumes could be cross-compared. For the ROI analysis, the two-run localizer data first entered the GLM. Then, the “Faces vs. Objects” contrast was applied, with the FFA defined individually and exclusively in the right hemisphere (FFA was located in the Talairach space of *X*: 40–50; *Y*: −45 to −55; *Z*: −10 to −20, with a corrected threshold of *p* < 0.5). The summed *t*-values and FFA-averaged PSC were extracted and compared with the earlier findings. Finally, for the multi-voxel pattern analysis in the searchlight fashion, the Princeton MVPA toolbox (https://pni.princeton.edu/pni-software-tools/mvpa-toolbox) and the CoSMoMVPA (https://www.cosmomvpa.org/) were implemented (Oosterhof et al., 2016), with the default setup, and averaged individual classification accuracy (commonly across any two conditions) maps into one-tailed *t*-maps (see uploaded results in NeuroVault).

## Results

### Behavioral results

**Figure 3:**
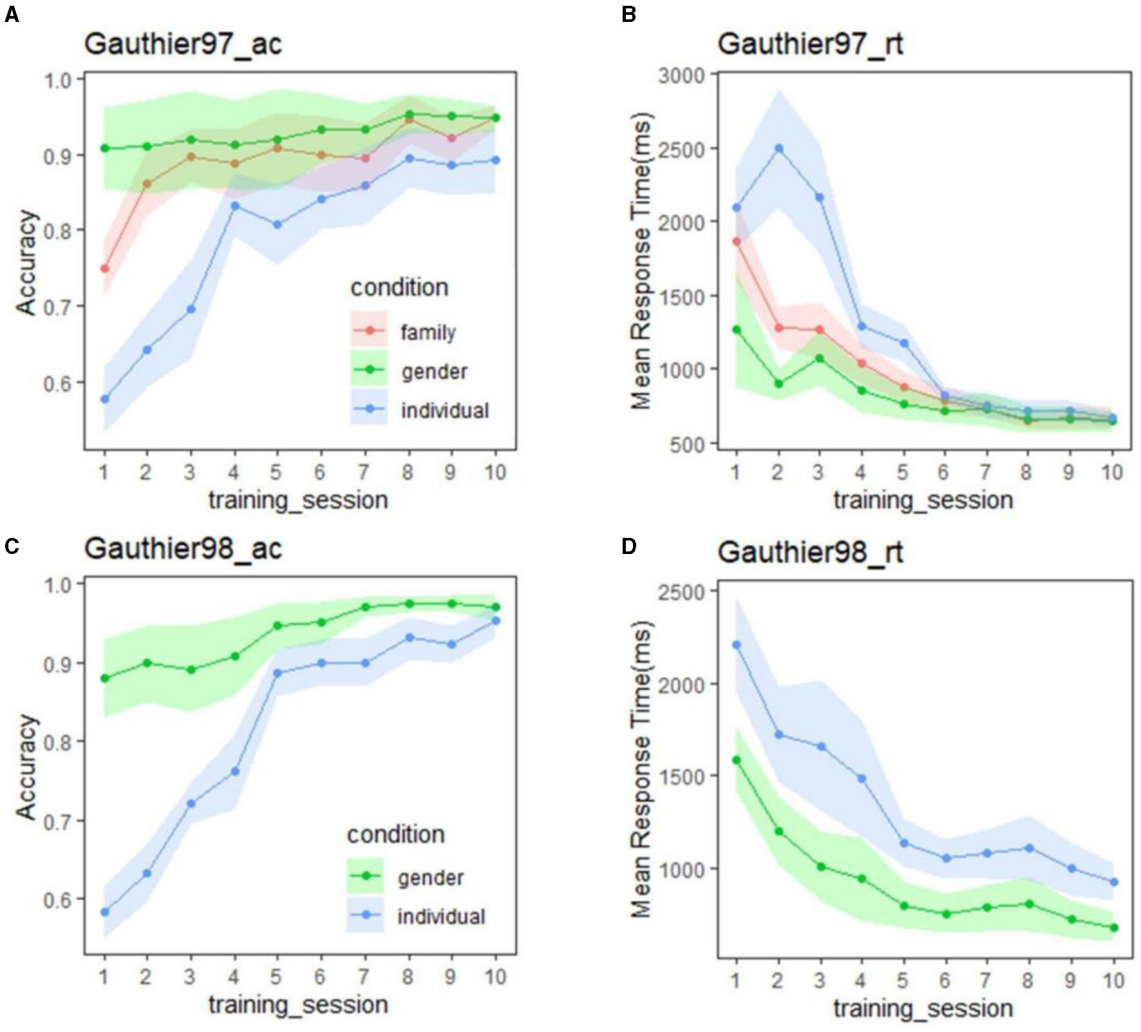
**(A)** Mean accuracy of Gauthier97 group (*n* = 8) for the three types of category level (gender, family, and individual levels) of the verification task; **(B)** Mean response time (in milliseconds) of Gauthier97 on the same verification task. The response time of three category levels was statistically insignificant from Day 4. **(C)** Accuracy of Gautheir98 group (*n* = 7) for the two types of category level (gender and individual levels). **(D)** Mean response time (in milliseconds) of Gauthier98 in the verification task. Note that the individual-level RT combined both named and unnamed Greebles and the similarity between our **(D)** and figure 3B (p. 3953) of Brants et al. (2011).

Figure 3 summarizes the mean accuracy (left panel) and RTs (right panel) by session results of the verification tasks in the two Greebles training regimes (Gauthier97 on top). For both Gauthier97 and Gauthier98, the gender accuracy was all around 90% after the first session [*F*_(1,14)_ = 0.145, *p* = 0.71, η^2^ = 0.01]. As for the accuracy at the family and the individual levels, participants all improved with training [comparing two groups' individual-level accuracy between the first and the last(tenth) session, *F*_(1,30)_ = 108.35, *p* <0.01, η^2^ = 0.78]. These accuracy results (Figures 3A, C) suggest that all participants could accurately identify Greebles on the designated (gender, family, or individual) level at the end of training.

In terms of RT, although in Figure 3B (Gauthier97) the mean response times in the individual level were longer than those in the family and the gender levels [the 1st ~5th session: *F*_(2,21)_ = 3.55, *p* = 0.047, η^2^ = 0.253], with training the RT difference became closer and finally converged from the sixth all the way to the 10th session [the last 5 *F*_(2,21)_ = 0.07, *p* = 0.929–0.932, η^2^ = 0.0066–0.0069]. According to the canonical definition of Greeble expertise (Gauthier and Tarr, 1997; Gauthier et al., 1997) (usually focusing on the statistical insignificance between verification RTs of family and individual levels), our Gauthier97 subjects have become experts since the 6th session and kept practicing for 4 more sessions.

In contrast with Figure 3B, the Gauthier98 RT results (Figure 3D) were quite different: not only that the mean RT was about 1,000 ms (compared to the mean final RT in Figure 3B, around 500 ms) but there also seemed no trend of convergence throughout Gauthier98 training. Strikingly, Figure 3D results were similar to the training results reported by Brants et al. (2011, figure 3b, p. 3953). Inspecting their reported procedure: “*Participants were trained following the procedure used by Gauthier et al. (**1997**)*.” (ibid, p. 3951), we found the match between Brants et al.'s figure 3b (p. 3953) and figure 3d (plus the mismatch with the figure 3b) of the current study, lending support to the idea that Brants et al. (2011) misquoted the Gauthier98 as Gauthier97 training paradigm. Their following methodological descriptions “*The participants learned the family labels and individual names for five Greebles in the first session and then learned five more Greebles in the following three sessions. Ten Greebles remained unknown throughout the train, which made the tasks more difficult*” (ibid., 3951) further verifying our suspicion (of their mis-adoption).

**Figure 4:**
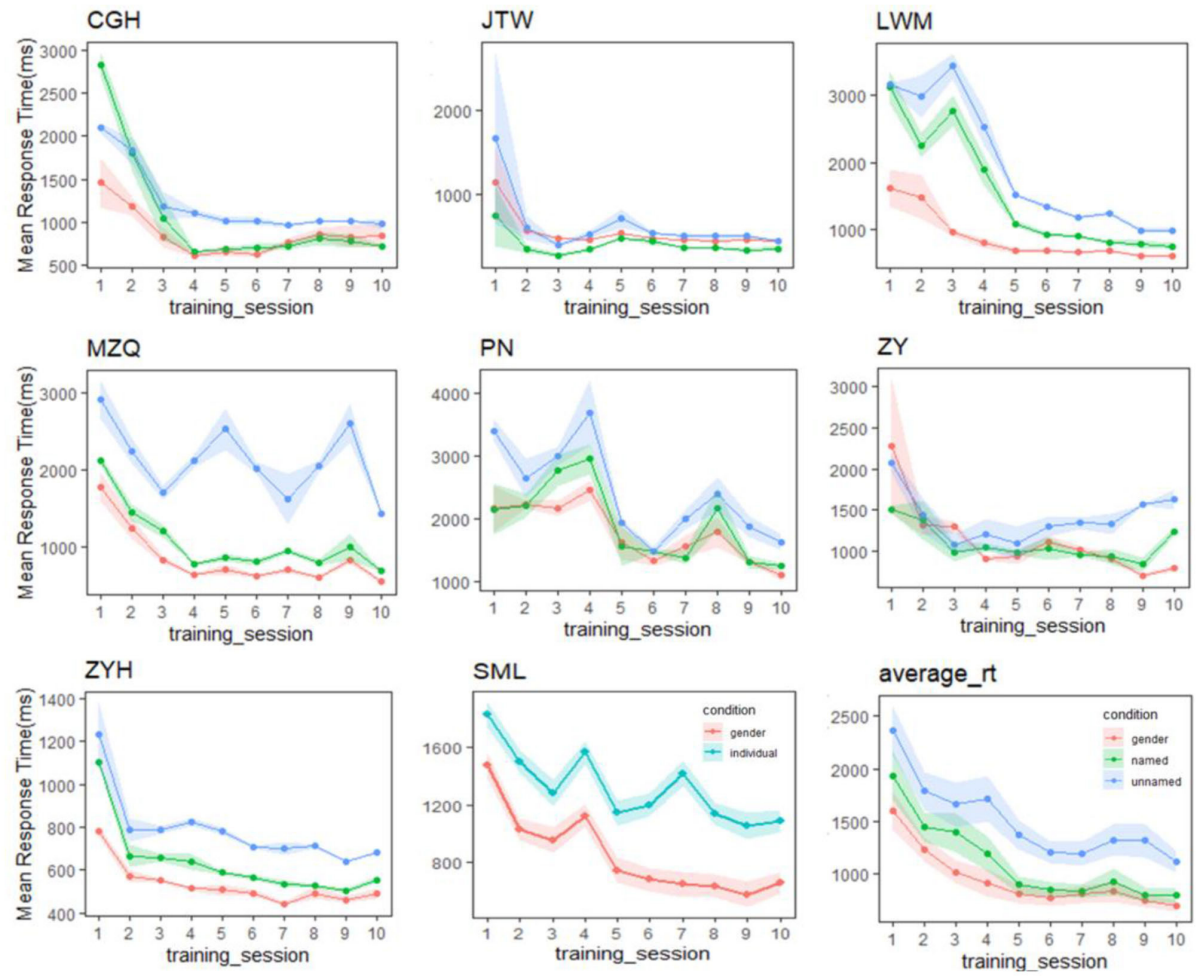
Performance in the verification task throughout training for each expert participant in the Gauthier98 group. While the individual-named Greebles RTs gradually converged with the gender RTs, our individual-unnamed Greebles also gradually decreased, but not converge, with training. One potential explanation was the individual variability inherent in the small samples. Overall, this inability to converge between the two levels renders the Gauthier98 training regime unfit for the behavioral training of Greeble experts.

Two follow-up observations remain. First, since our Gauthier98 results mimicked those of Brants et al. (2011), how about the replications between our Gauthier98 results and those from the original Gauthier et al. (1998), especially the gender and named/unnamed individual-level training RTs? For this comparison (figure 4b, ibid, p. 2407; and the similar plot of the current study, shown in the lower right corner of Figure 4), one sees a slightly different picture: While the individual-named Greebles RTs gradually converged with the gender RTs in both studies [similar to what was found in Gauthier et al. (1998)], our individual-unnamed Greeble RT, while also not converged with the mean gender RT, was gradually decreased with training [unlike what was shown in figure 4b of Gauthier et al. (1998), where the unnamed RT was constantly high]. Further comparison between our individual training RT plots (Figure 4), alongside the similar figure 5 of Gauthier et al. (1998, p. 2408), revealed that one likely explanation was the individual variability inherent in the small samples of both the original (*N* = 12) and the current replicated Gauthier98 (*N* = 8) study, respectively. Overall, both studies shared the converging finding between the gender and the individual-named RTs but no convergence between the gender and individual-unnamed RTs [figure 4 of Gauthier et al. (1998), and lower right of Figure 4 in this study]. This inability to converge between the two (here individual-combined and gender) levels, a signature of perceptual expertise (Tanaka and Gauthier, 1997), renders the Gauthier98 training regime relatively unfit for the behavioral training of Greeble experts.

Second, there was an expertise-reaching RT difference between the two training regimes: In Gauthier97, it was ~500 ms at the end of expertise training, whereas in Gauthier98, the end RT hovered ~1,000 ms. Such a two-fold RT difference (500 ms in Gauthier97 vs. 1,000 ms in Gauthier98) likely resulted from the additional processing steps in Gauthier98. Figure 11 (in discussion) lists two of the possible stages of such putative decisions Greeble trainees faced during the training processes: one upon the label presentation and another upon Greeble presentation. With the additive factors logic (Sternberg, 1969), the 500-ms gap between the two training regimes could be reasonably explained.

**Figure 5:**
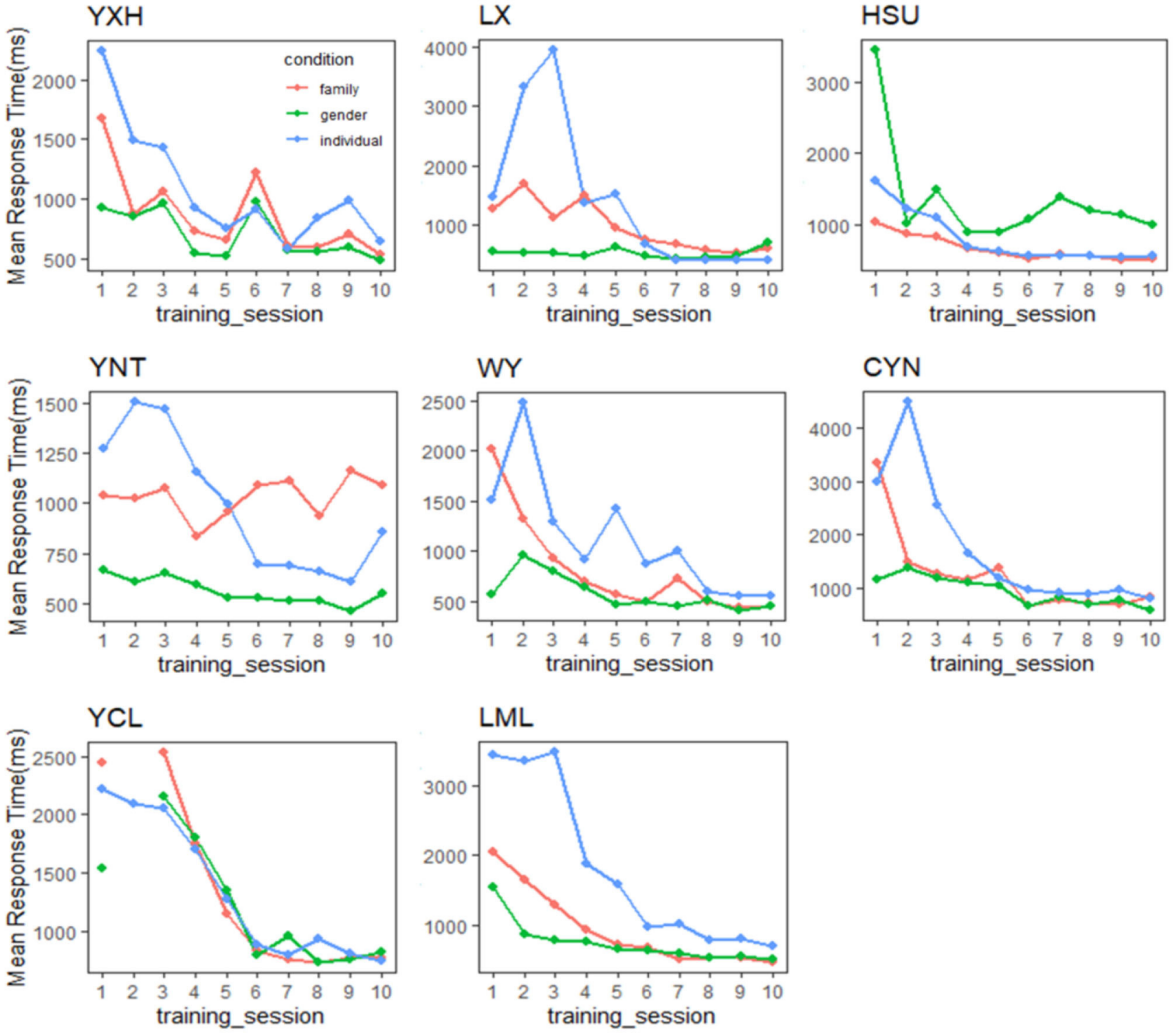
Performance in the verification task throughout training for each expert participant in the Gauthier97 group. Convergence happened among the trends of the mean response time of gender, family, and individual name. Only in the Gauthier97 group, and only after training, which reflects modulation of the expertise category following the “appropriate” training regime.

In sum, the converging RT results with Gauthier97 training, as shown in Figure 5, in contrast to the no-converging results with the Gauthier98 paradigm, suggest that only the Gauthier97 group, and only after training, were the participants successfully achieving the criterion of perceptual expertise. We, therefore, concluded that the expertise training paradigm of Gauthier97 was more robust and, therefore, a more “appropriate” training regime (than that of Gauthier98, or Brants et al., 2011) in generating Greeble experts.

### fMRI results: ROI (FFA) analyses

To distinguish different accounts of the FFA activities, namely the perceptual expertise vs. the face likeness, three proposed ROI (FFA) analyses were tested here, all between Gauthier97 and Gauthier98 training regimes: (a). the comparisons among upright faces, objects, and Greebles, between before and after training; (b). the neuronal inversion effect (NIE): the upright vs. inverted Greebles and faces, across before, during, and after training; and (c). the neural adaptation effects for both faces and Greebles, again before vs. after training. The perceptual expertise (PE) hypothesis of the FFA predicts that (a) FFA responses to Greebles would increase significantly from the before to after training, but no such effect for faces or objects, and only be so in the appropriate (e.g., Gauthier97) training paradigm; and (c) a significant increase in Faces after Greebles (e.g., neural adaptation) only after training, and under the appropriate, or Gauthier97, paradigm, as well. In contrast, the FFA face-specificity hypothesis, according to Brants et al. (2011), would predict: (b) an NIE for faces both before and after training, but not for asymmetric Greebles (given their much less, if not zero, face likeness), given the premise that NIE being a consistent and reliable index of face-related processing. This assumption will be examined in the funnel plot of the extant literature.

#### ROI (FFA) analysis

**Figure 6:**
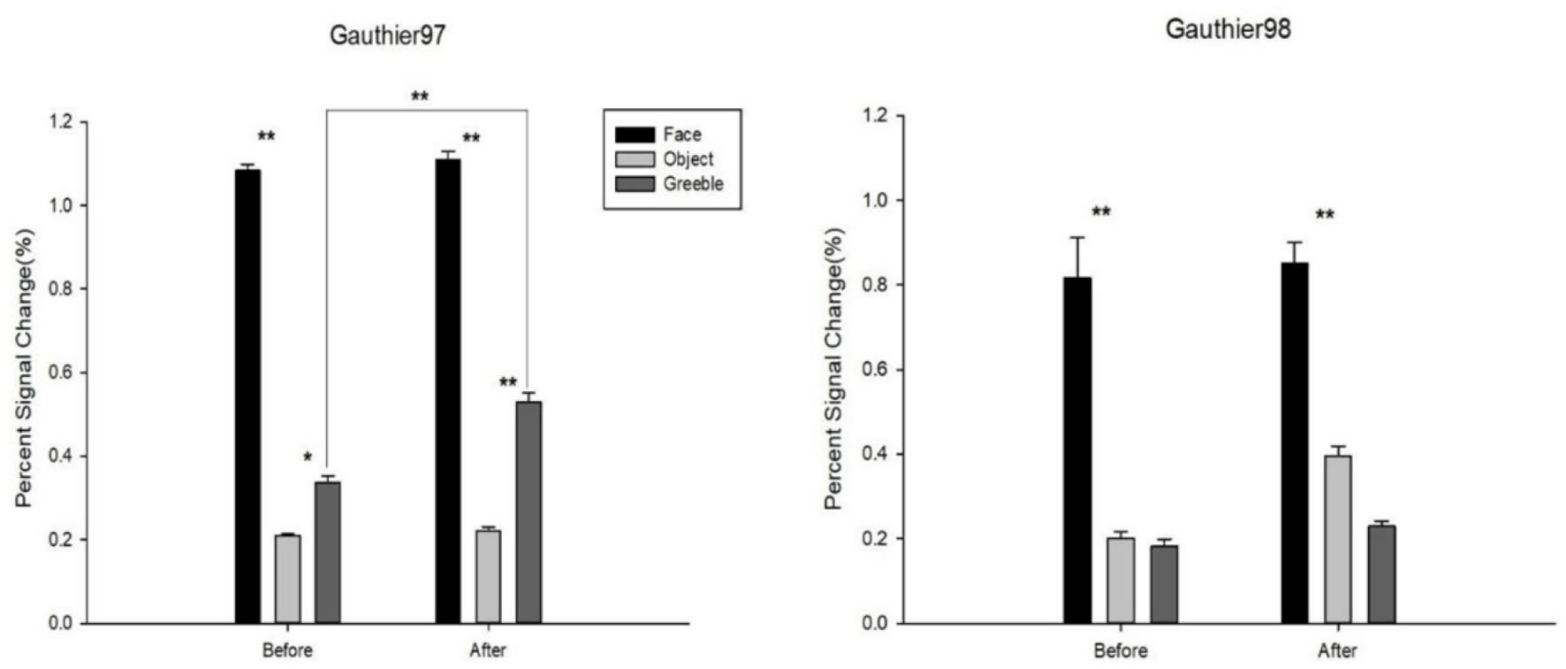
Percent signal changes for faces, objects, and Greebles in the FFA. Error bars were standard errors. Shown on the left by Gauthier97 training results, right by Gauthier98. *N* = 8 in each training group. As shown, there was a significant training effect for Greebles in the Gauthier97, but not in the Gauthier98, paradigm. * means *p* < 0.05, and ** means *p* < 0.01, the 95% confidence interval.

The results of FFA activities for faces, objects, and Greebles before and after training, as well as for both Gauthier97 and Gauthier98, are shown in Figure 6. For the “Faces vs. Objects” comparison, the main effects were found for both before [*F*_(1,110)_ = 301.29, *p* < 0.01] and after training [*F*_(1,110)_ = 195.64, *p* < 0.01]. No training effect (after vs. before training) was found for faces in the FFA [*F*_(1,110)_ = 0.1353, *p* = 0.71]. Similarly, the comparison of “Greebles vs. Objects” yielded a main effect both before [*F*_(1,110)_ = 6.17, *p* = 0.014] and after training [*F*_(1,110)_ = 22.97, *p* < 0.01]. More importantly, in the Gauthier97 paradigm, there was a significant difference for Greeble activations before vs. after training [*F*_(1,110)_ = 7.31, *p* < 0.01], indicating the Greeble training effect on the FFA (see Figure 6 left).

In contrast, as shown in Figure 6 right, other than the similarly significant “Faces vs. Objects” main effects both before [*F*_(1,110)_ = 26.06, *p* < 0.01] and after training [*F*_(1,110)_ = 21.12, *p* < 0.01], Gauthier98 results showed no “Greebles vs. Objects” main effects across training (both *p* > 0.05). Because the current training regimes strictly adhered to those in both Gauthier et al. (1999) and Brants et al. (2011), with the only exception the choice of asymmetric Greebles, these results were more in line with the perceptual expertise account.

#### Neural inversion effect in the FFA

**Figure 7:**
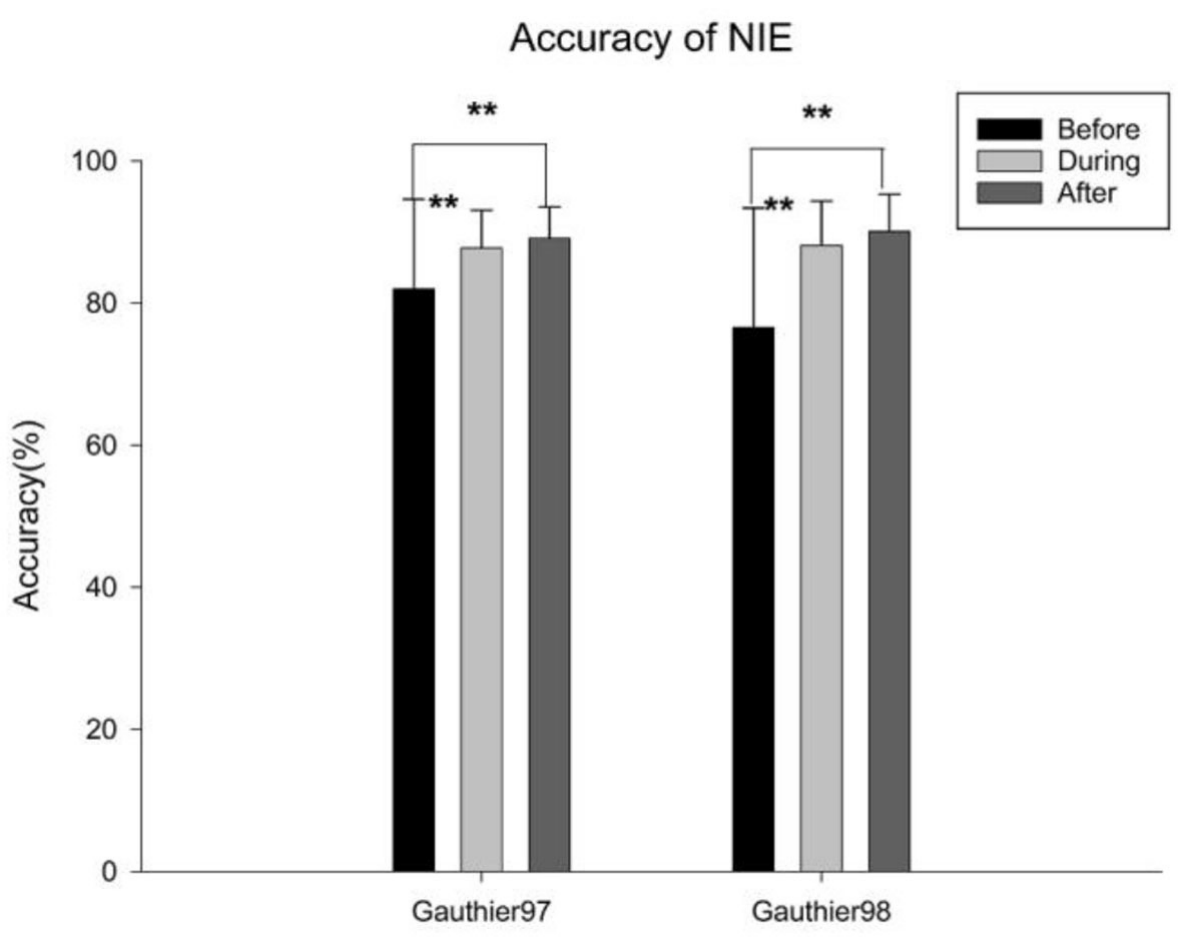
Mean correct rates of the identity-matching task, either upright or inverted faces or Greebles, across three fMRI scan sessions. Error bars represent standard errors. ** means *p* < 0.01, the 95% confidence interval.

The behavioral performance of all participants in the sequential matching task during the three fMRI sessions was shown in Figure 7. Both groups demonstrated improvements after five sessions of training [before vs. after training: Gauthier97: *F*_(1,78)_ = 7.1324, *p* < 0.01; Gauthier98: *F*_(1,78)_ = 16.68405, *p* < 0.01], different from the results reported by Brants et al. (2011).

**Figure 8:**
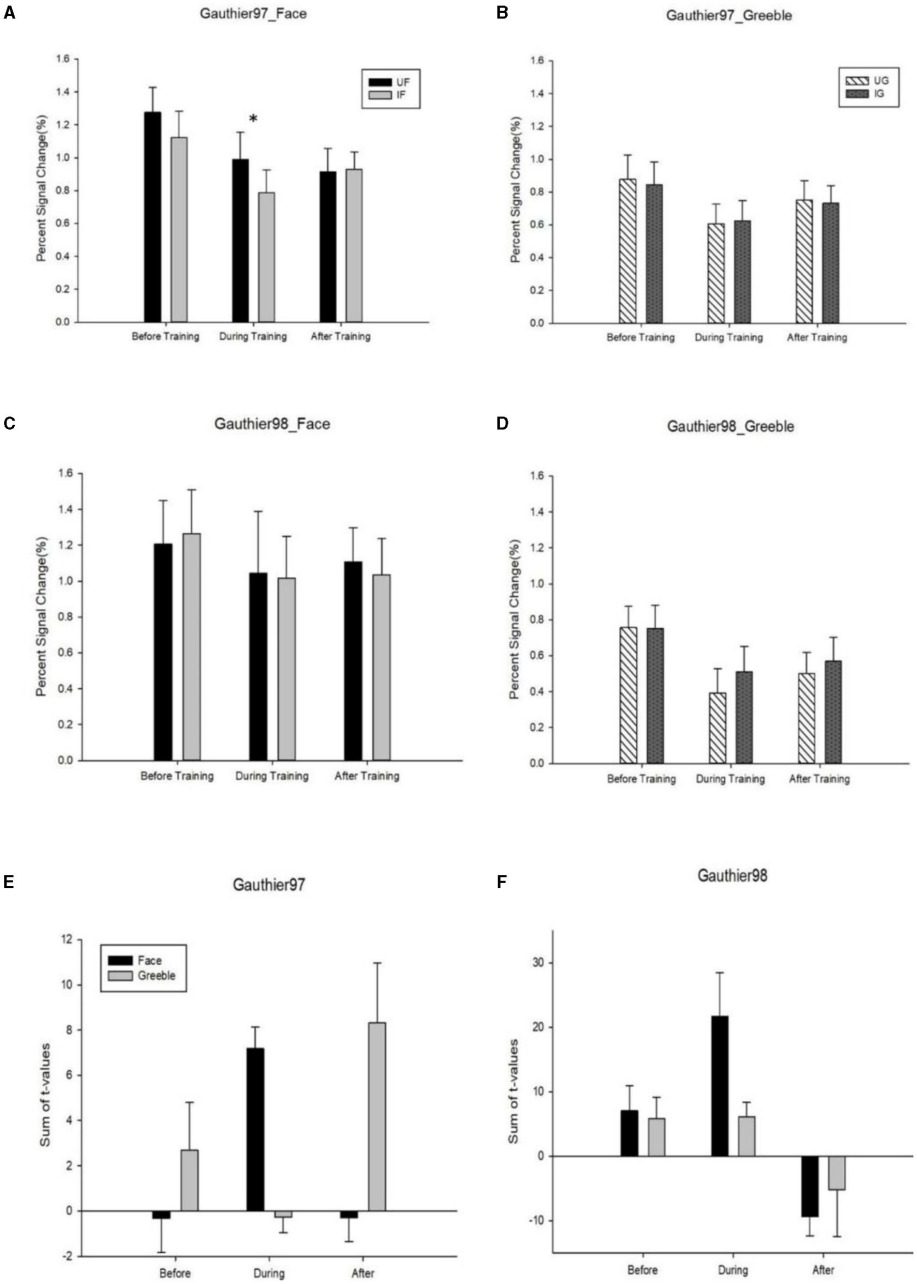
**(A)** FFA activities (percent signal change, or PSC) to the upright vs. inverted faces; **(B)** the same FFA neural inversion effect (or NIE) for Greebles; both **(A, B)** were from Gauthier97 paradigm; **(C)** FFA NIE for faces; **(D)** for Greebles; **(C, D)** were from Gauthier98 paradigm; **(E, F)** the summed *t*-values of NIE. *T*-values were summed and averaged across all FFA voxels corresponding to the upright vs. inverted stimulus version **(E)** for Gauther97, and **(F)** for Gauthier98. For all graphs, error bars denote standard errors. * means *p* < 0.05, the 95% confidence interval.

The mean Percent Signal Change (PSC) difference between the upright and the inverted faces, or Greebles, was defined as the neural inversion effect, or NIE. For faces, no significant NIEs were observed in either Gauthier97 or 98 (Figures 8A, C), across all three scan sessions (before, during, and after training). Out of the six possible face FIEs (three for each training group), only one (the during training of Gauthier97) was significant [*F*_(1,126)_ = 7.83, *p* = 0.0059]. As for Greebles, none of the six NIEs was significant (see Figures 8B, D). These results sharply contrast with Brants et al. (2011), who reported significant FIEs for faces and Greebles, both before and after training (four out of four). In other words, while the present study only found one significant NIE out of 12 possible tests (8.3%), Brants et al. got four out of four (or 100%) significant NIEs!

Although the PSC is now more pervasively used in the fMRI papers, the summed *t*-values, originally reported in Gauthier et al. (1999), might be another possible index to verify NIE. This method sums the *t*-values from each participant's FFA voxels and then averages across upright minus inverted conditions (and across subjects). As each participant's FFA contained different numbers of voxels, our summed *t*-values for faces and Greebles, again across three sessions, showed no significant interactions (though with trends of increase for Greebles NIE, in Gauthier97; see Figures 8E, F).

#### Meta-analysis of NIE

One untested assumption in supporting the NIE as the index of the “face specificity of FFA” account is its reliability: To be a strong indicator of face-related processing, NIE has to be consistently identified in relevant literature whenever upright and inverted faces are compared. To find out, we performed a search with the keywords “FFA” and “neural inversion effect,” which yielded 18 fMRI articles matching the criteria of “fMRI studies containing FFA NIE results.” As shown in Table 1, seven articles identified positively significant NIEs, two articles found negatively significant NIEs (i.e., FFA activities for inverted faces higher than those for upright faces), and the remaining 10 studies (nine plus the present study) showed no significant NIE for faces at the FFA.

**Figure 9:**
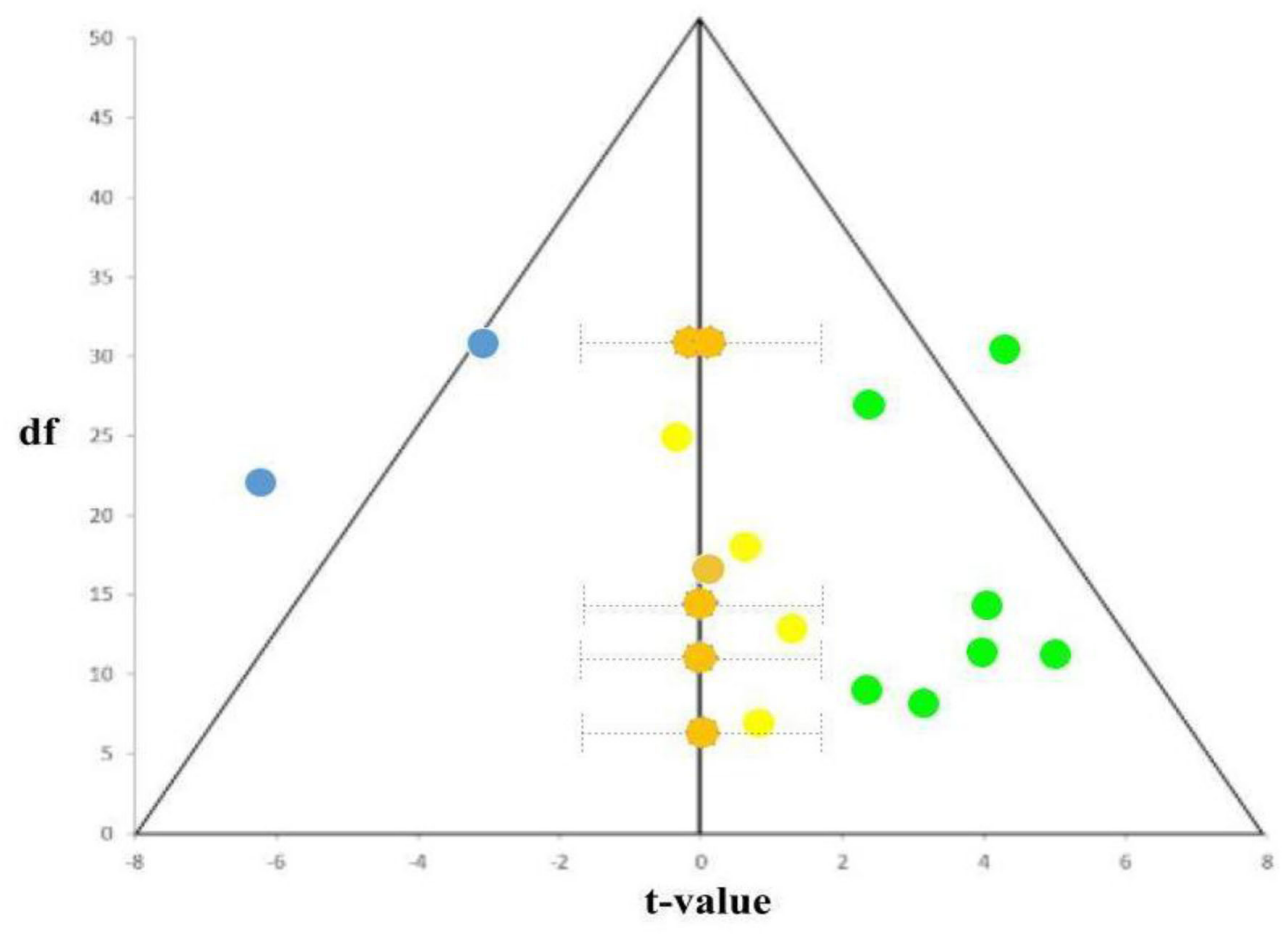
Funnel plot of reported *t*-statistics from Table 1. The positively significant NIEs were colored in green (seven studies), no significant NIEs in yellow (reported; five studies) and orange (estimated from confidence intervals; also five studies), and negative NIEs in blue (two studies). As shown, the almost 1:1 (10: 9) ratio between significant (including both positive and negative NIEs) and not-significant (including both reported, light-yellowed dots and unreported/estimated, dark-yellowed dots, NIEs) studies support not just the existence of publication bias (i.e., only significant *t*-values were reported), but also the unreliability of NIE as the index of face selectivity (~ = majority of studies with NIE in the FFA should be positively significant).

A funnel plot of these 19 studies (14 with reported stats) is also shown in Figure 9. Five studies reporting non-significant results were given estimated *t*-values and 95% confidence intervals. With these results, one can see that the NIEs in the FFA did suffer certain degrees of publication bias: the slightly more no difference (Grimaldi et al., 2016) than positively (Kanwisher, 2000) and negatively (Sugita, 2008) significant NIE in the FFA in the literature, seriously undermining its reliability assumption that if NIE is a face-related index, most if not all, published studies should yield unanimously positive and significant NIE in the FFA for faces.

Finally, the last issue with Brants et al.'s reasoning is that when faces show reliable NIE (which is not the case, as it turns out), finding NIE (as they demonstrated in Brants et al., 2011) would reversely imply that the stimulus should be faces (or face-like). Such reasoning is exactly reverse inference (Poldrack, 2006), a situation that neuroscientists should try to circumvent. The Bayes theorem could estimate the probability of *p*(faces | NIE) = *p*(faces)^*^p(NIE | faces)/*p*(NIE). With the help of the neurosynth database, which contains = 896 (papers with the term “faces”)/14,371 (total number of published papers in the neurosynth database, as of Jul 10, 2023) ^*^ 7/20 (the probability that was acquired by the meta-analysis below)/1/3 (NIE could be either positive, no difference, or negative) = 0.06, or around 1 in 20 studies. These results once again suggest the fallibility of equating NIE as face(like) processing.

#### Neural adaptation effect in the FFA

**Figure 10:**
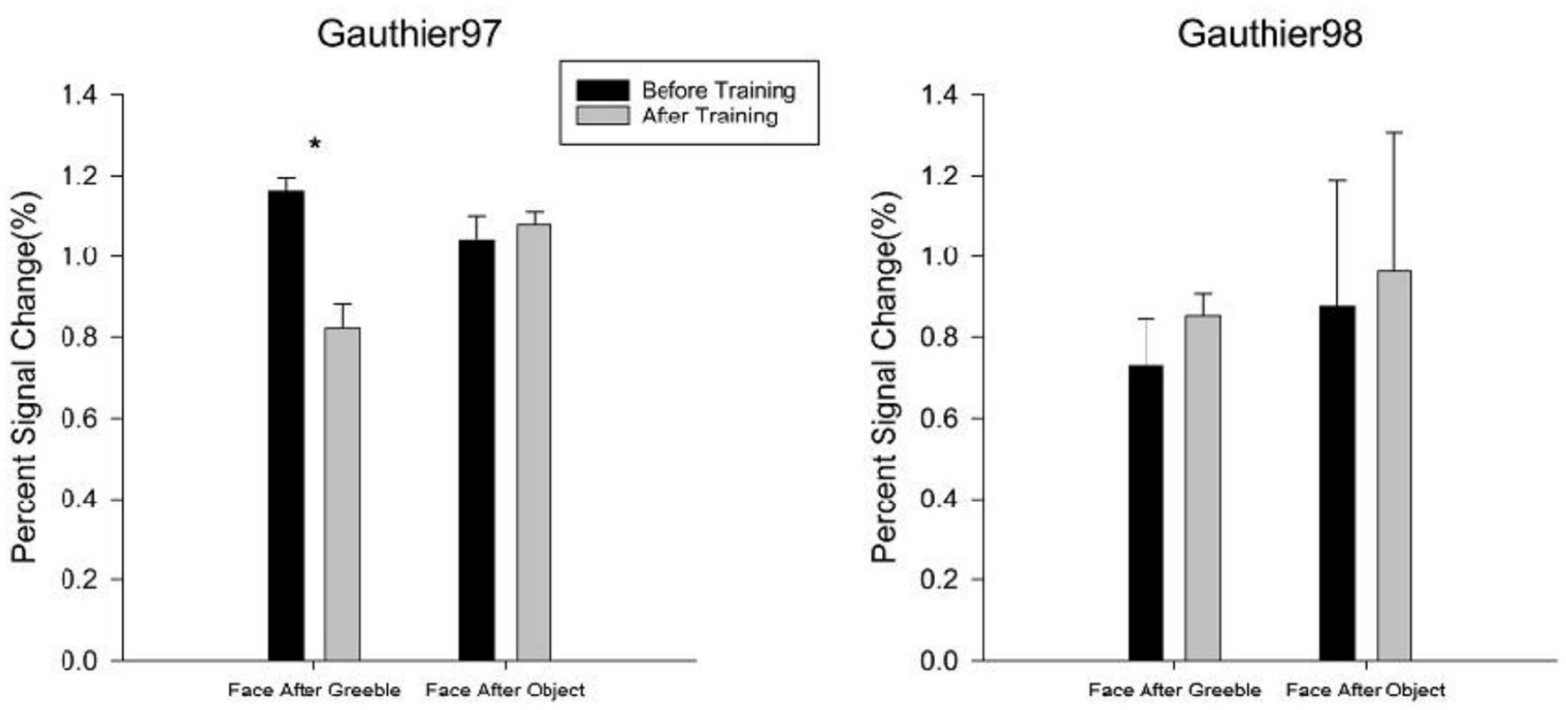
Neural adaptation effect in the FFA. The comparisons of percent signal change for “Faces after Greebles” vs. “Faces after Objects” in both Gauthier97 (*N* = 8) vs. Gauthier98 (*N* = 8) training regime were taken as the evidence for the support of the perceptual expertise hypothesis of FFA. Error bars represent standard errors. * means *p* < 0.05, the 95% confidence interval.

Another index of successful training would be to see whether the beholders' FFA responses for faces, after becoming Greeble experts, will become weaker (aka. adapted) following the presentation of Greebles, compared to the control condition of the before-training session (where Greebles are not face-like to FFA yet) and also compared to the inappropriate training condition (e.g., Gauthier97 vs. Gauthier98). Other than the two abovementioned controlled comparisons, there is one more specific prediction: the neural adaptation would only happen to the faces in the “Faces after Greebles” condition (not the other way around, i.e., “Greebles after Faces,” because Greebles would always be adapted following faces presented to the FFA, which is constantly face-selective), compared to the controlled “Faces after Objects” condition. Figure 10 shows *t*-test comparison results for both the before vs. after training sessions and the Gauthier97 and Gauthier98 regimes.

For the Gauthier97 group, there was a significant difference in the “Faces after Greebles” condition [*t*_(7)_ = 2.01, *p* = 0.04], in line with the prediction that training-induced Greeble expertise recruiting face-like responses in the FFA, thereby making the subsequent faces “adapted.” In contrast, the “Faces after Objects,” plus neither “Faces after Greebles” nor “Faces after Objects” comparisons in the Gauthier98 group, showed any adaptation effects in the FFA. Together, these four comparison results strongly agree with the predictions made by the perceptual expertise account of the FFA.

#### fMRI results: MVPA classification performances

In light of the growing prevalence of multi-voxel pattern analyses, we also compared the category classification performances between Gauthier97 and Gauthier98, and between before vs. after training, using both ROI (e.g., FFA) and the whole-brain searchlight analysis fashion. The reason why multi-voxel pattern analyses were reported here was that in addition to the planned univariate analyses between upright and inverted faces and Greebles (and between training regimes), multivariate fMRI analyses have also received popularity and provided additional insights into the neural representation under investigation. For the current study, it may be of interest to check whether the “Greebles vs. Objects” contrast has become easier to classify (and “Faces vs. Greebles” harder to classify) after training, since Greeble training has increased FFA's sensitivity to Greebles, making Greebles more face-like (or less object-like), and probably also only in the more appropriate Gauthier97 training regime.

As shown in Table 2, the results of accuracy, recall, precision, F1-score and area under the ROC curve are in general agreement. Therefore, the recall/precision/F1-score results were aggregated for paired *t*-comparisons. The results show that there is a significant difference in recall (*t*_(7)_ = −2.758, *p* = 0.028; Cohen's *d* = 0.98) and F1-score (*t*_(7)_ = −2.749, *p* = 0.029; Cohen's *d* = 0.97) for Gauthier97 group before vs. after training, and the precision with marginal significance (*t*_(7)_ = −2.086, *p* = 0.075). The increase in recall in the Gauthier97 group indicates that as individuals become Greeble experts, their FFA sensitivity to Greeble (e.g., true positives) has increased. Additionally, since the F1 score is related to both recall and precision, the change in F1 differences signifies that with individual Greeble training, the FFA performs better in classifying faces and Greebles.

**Table 2:** ROI MVPA analysis for 2 (Gautiher97 vs. Gauthier98) ^*^2 (before_ vs. after training) ^*^2 (“Greeble vs. face” and “Greeble vs. object”) conditions (*n* = 8 in each condition).

**Before training**										**Before training**									
**Greeble vs. face individual FFA**					**Leave 1 trial out, linear kernel, C** = **1, LIBSVM**					**Greeble Vs. object individual FFA**					**Leave 1 trial out, linear kernel, C** = **1, LIBSVM**				
**Gauthier97**	**CYN**	**WY**	**LML**	**LX**	**XHJ**	**YXH**	**YNT**	**LYJ**	**Average**	**Gauthier97**	**CYN**	**WY**	**LML**	**LX**	**XHJ**	**YXH**	**YNT**	**LYJ**	**Average**
Average	0.5	0.5	0.5625	0.625	0.4375	0.375	0.75	0.1875	0.49219	average	0.5625	0.6875	0.5	0.3125	0.875	0.5625	0.75	0.125	0.54688
Recall	**0.375**	**0.5**	**0.625**	**0.625**	**0.625**	**0.25**	**0.75**	**0.125**	**0.48438**	recall	0.5	0.625	0.625	0.25	0.875	0.625	0.75	0.25	0.5625
Precision	0.5	0.5	0.5556	0.625	0.4545	0.3333	0.75	0.1429	0.48266	precision	0.5714	0.7143	0.5	0.2857	0.875	0.5556	0.75	0.2	0.5565
f1_score	**0.4286**	**0.5**	**0.5882**	**0.625**	**0.5263**	**0.2857**	**0.75**	**0.1333**	**0.47964**	f1_score	0.5333	0.6667	0.5556	0.2667	0.875	0.5882	0.75	0.2222	0.55721
AUC	0.5	0.5	0.5625	0.625	0.4375	0.375	0.75	0.1875	0.49219	AUC	0.5625	0.6875	0.5	0.3125	0.875	0.5625	0.75	0.125	0.54688
**Gauthier98**	**LWM**	**PN**	**LSM**	**ZY**	**JTW**	**MZQ**	**CGH**	**ZYH**	**Average**	**Gauthier98**	**LWM**	**PN**	**LSM**	**ZY**	**JTW**	**MZQ**	**CGH**	**ZYH**	**Average**
Average	0.875	0.375	0.625	0.625	0.875	0.1875	0.6875	0.75	0.625	Average	0.875	0.25	0.375	0.6875	0.4375	0.5625	0.625	0.37	0.52344
Recall	0.875	0.25	0.75	0.625	1	0.25	0.625	0.875	0.65625	Recall	1	0.25	0.25	0.75	0.375	0.625	0.625	0.375	0.53125
Precision	0.875	0.3333	0.6	0.625	0.8	0.2222	0.7143	0.7	0.60873	Precision	0.8	0.25	0.3333	0.6667	0.4286	0.5556	0.625	0.375	0.50428
f1_score	0.875	0.2857	0.6667	0.625	0.8889	0.2353	0.6667	0.7778	0.62764	f1_score	0.8889	0.25	0.2857	0.7059	0.4	0.5882	0.625	0.375	0.51484
AUC	0.875	0.375	0.625	0.625	0.875	0.1875	0.6875	0.75	0.625	AUC	0.875	0.25	0.375	0.6875	0.4375	0.5625	0.625	0.375	0.52344
**After training**										**After training**									
**Greeble vs. face individual FFA**					**Leave 1 trial out, linear kernel, C** = **1, LIBSVM**					**Greeble vs. object individual FFA**					**Leave 1 trial out, linear kernel, C** = **1, LIBSVM**				
**Gauthier97**	**CYN**	**WY**	**LML**	**LX**	**XHJ**	**YXH**	**YNT**	**LYJ**	**Average**	**Gauthier97**	**CYN**	**WY**	**LML**	**LX**	**XHJ**	**YXH**	**YNT**	**LYJ**	**Average**
avaerage	0.5625	0.625	0.625	0.5625	0.8125	0.4375	0.6875	0.3125	0.57813	Avaerage	0.75	0.5	0.5625	0.375	0.6875	0.25	0.75	0.375	0.53125
recall	**0.75**	**0.625**	**0.625**	**0.75**	**0.875**	**0.5**	**0.625**	**0.375**	**0.64063**	recall	0.75	0.625	0.5	0.375	0.75	0.25	0.75	0.375	0.54688
precision	0.5455	0.625	0.625	0.5455	0.7778	0.4444	0.7143	0.3333	0.57635	precision	0.75	0.5	0.5714	0.375	0.6667	0.25	0.75	0.375	0.52976
f1_score	**0.6316**	**0.625**	**0.625**	**0.6316**	**0.8235**	**0.4706**	**0.6667**	**0.3529**	**0.60336**	f1_score	0.75	0.5556	0.5333	0.375	0.7059	0.25	0.75	0.375	0.53685
AUC	0.5625	0.625	0.625	0.5625	0.8125	0.4375	0.6875	0.3125	0.57813	AUC	0.75	0.5	0.5625	0.375	0.6875	0.25	0.75	0.375	0.53125
**Gauthier98**	**LWM**	**PN**	**LSM**	**ZY**	**JTW**	**MZQ**	**CGH**	**ZYH**	**Average**	**Gauthier98**	**LWM**	**PN**	**LSM**	**ZY**	**JTW**	**MZQ**	**CGH**	**ZYH**	**Average**
Average	0.5625	0.6875	0.6875	0.6875	0.75	0.8125	0.375	0.8125	0.67188	Average	0.6875	0.9375	0.375	0.6875	0.625	0.75	0.375	0.9375	0.67188
Recall	0.5	0.75	0.75	0.75	0.75	0.875	0.375	0.75	0.6875	Recall	0.75	0.875	0.375	0.625	0.25	0.75	0.375	0.875	0.60938
Precision	0.5714	0.6667	0.6667	0.6667	0.75	0.7778	0.375	0.8571	0.66643	Precision	0.6667	1	0.375	0.7143	1	0.75	0.375	1	0.73513
f1_score	0.5333	0.7059	0.7059	0.7059	0.75	0.8235	0.375	0.8	0.67494	f1_score	0.7059	0.9333	0.375	0.6667	0.4	0.75	0.375	0.9333	0.6424
AUC	0.5625	0.6875	0.6875	0.6875	0.75	0.8125	0.375	0.8125	0.67188	AUC	0.6875	0.9375	0.375	0.6875	0.625	0.75	0.375	0.9375	0.67188

In addition to the original classification accuracy, recalls, precisions, F1-scores, and area under the ROC curves (AUC, which matches exactly the classification accuracy) are also provided. Bold-typed texts were with significant results (p < 0.05).

In line with our univariate analysis results, the increased Greeble sensitivity (indexed by the recall score) and increased Greeble-Face separability (by the F1-score), only in the Gauthier97 paradigm, once again partially support the clear modulation of appropriate training on subjects' representations. By partially, we mean that the evidence was not indexed in classification accuracy, which may be due to the variability inherent in various dependent measures. It may also be that the choice of to-be-compared stimuli was relevant since both studies showed higher classification accuracies in chess experts' FFA on chess boards (Bilalić et al., 2011), and for radiologists' FFA on chess X-rayed pictures (Bilalić et al., 2014), the control stimuli were all scene pictures. Future analysis could compare the effects of training between Greebles to various control stimuli (i.e., the scene condition in our Peelen localizer scan). In addition, the increased F1-score may also be compatible with the interaction between faces and Greebles in the FFA, reflected by the adaptation effect of FFA for faces after Greebles (see Figure 10) after training. Taken together, we can summarize that the naive “before training, Greebles are object-like; after training, Greebles are face-like” hypothesis may not be directly applicable to multivariate analysis, as evidenced by our current MVPA analysis results of increased face vs. Greeble sensitivity.

## Discussion

The present study revisited two assumptions behind Brants et al. (2011) JOCN article, one of the seminal studies in the prolonged discussions for vs. against the perceptual expertise of FFA and one widely known debate in modern cognitive neuroscience. As one of the oft-cited stimuli in the field of perception neuroscience, Greebles was also seen in many textbooks of vision research (Wolfe, 2009; Ward, 2015). The claim that the training-induced FFA activity increases were primarily due to Greebles' face likeness, instead of the original putative expertise training, indeed raises concern about the premises behind these claims: that (1) Brants et al. (2011) should have also replicated the Greeble training effects, at least behaviorally; and (2) that the neural inversion effect, or NIE at the FFA, should be a reliable index of human face processing. But as the current study has demonstrated, the first premise was unsubstantiated that by differentiating the Greeble training paradigm as the Gauthier97 vs. Gauthier98 version, the session-wise RT of the verification task between the two paradigms (Figure 3) was drastically different, suggesting that Brants et al. (2011) adopted the Gauthier98 paradigm (by their highly similar RT patterns). In contrast, the original Greeble training study (i.e., Gauthier et al., 1999) adopted the Gauthier97 version. Further individual RT analysis in Gauthier98 (e.g., Figure 5) showed that the large variability among participants was the main reason behind the inability to converge between RTs for trained and untrained Greebles, the main difference between the original Gauthier98 training results and those of the current study.

In addition, the current study further provided three pieces of the fMRI evidence that are consistent with the experience modulation account of the FFA: (a) FFA activities to Greebles after training were significantly larger than that of the before training condition, only under the appropriate (i.e., Gauthier97) training condition; (b) the lack of NIE effect in the FFA, the second premise for the Greebles are face-like hypothesis, plus the inconsistency of NIE effects at FFA in the extant literature (seven positives, two negatives, and 10 no activation differences between upright and inverted faces) further wrecked the assumption of the Brants et al. (2011) claim (that NIE in the FFA is the reliable index of human face processing); and (c) most importantly, the FFA adaptation effect, specifically for faces after Greebles, but not for faces after objects, and also not vice versa (i.e., Greebles after faces); under the appropriate (i.e., Gauthier97) regime, and only after (not before) training. Altogether, this combined evidence, including the clear RT difference by training paradigm and the close similarity between that of Gauthier98 (Figure 3D) and Brants et al. (2011) on the behavioral side and the three fMRI univariate results (FFA training effect, no clear NIE in the FFA, and FFA adaptation effect) on the neuronal side, is jointly in favor of the perceptual experience account and incongruent with the Greebles-like-face account, put forward by the Brants et al. (2011) study.

**Figure 11:**
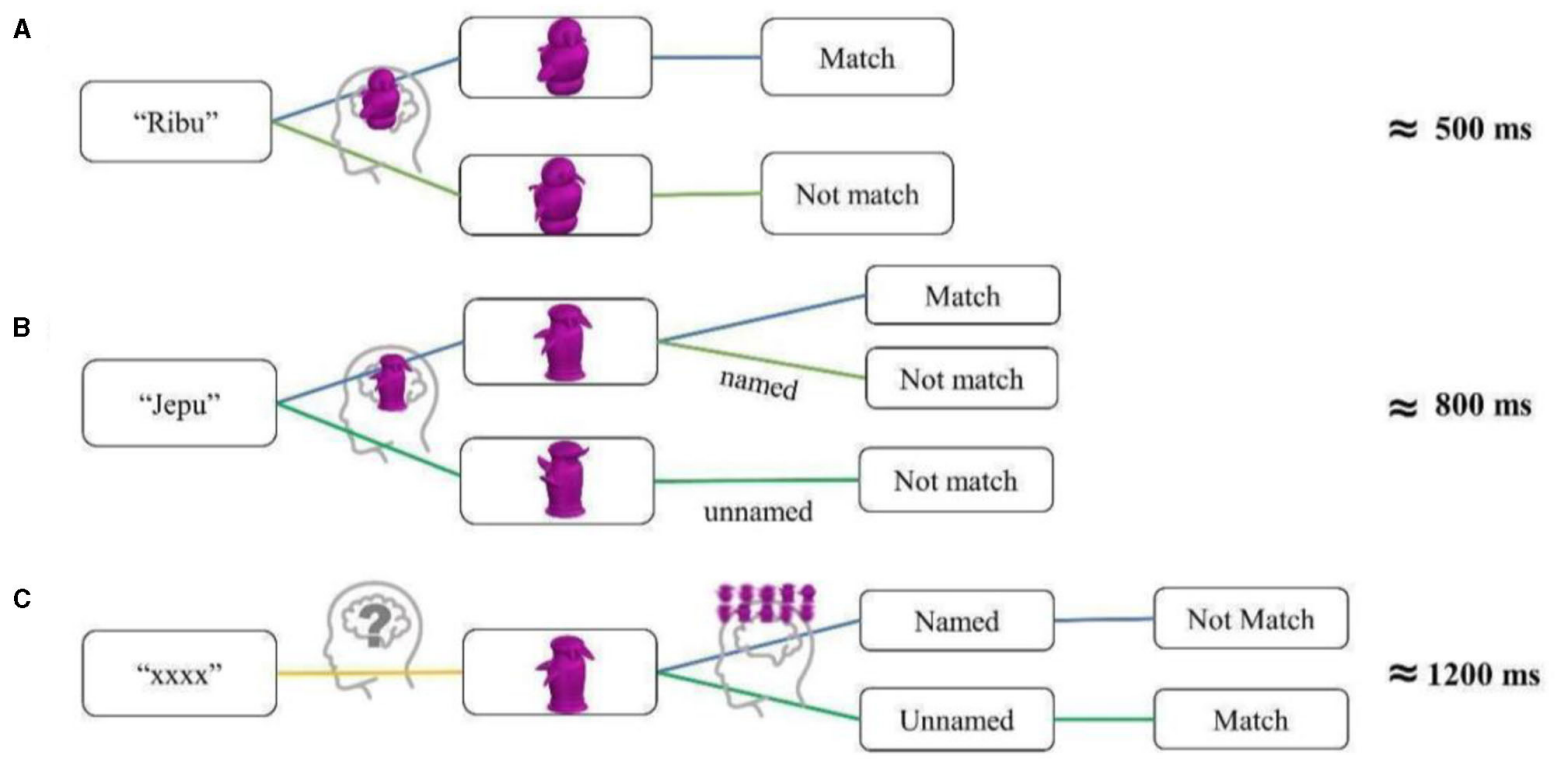
**(A)** Proposed task flow of the verification task in the Gauthier97 training paradigm. **(B, C)** The same flowchart for the Gauthier98 training paradigm, for named and unnamed trials, respectively. The proposed steps in each condition (representing the putative stages of processing) proportionally correspond to the average response times at the end of the training: Gauthier97: 500 ms, Gauthier98 named trials: 800, and unnamed trials: 1,200 ms. These differences may explain why Gauthier97 is the relatively appropriate paradigm for Greeble training effects.

The results of ROI MVPA and searchlight analysis on various combinations of categories, training sessions, and regimes, as revealed in both Figure 11 and https://neurovault.org/collections/13893/, were not in line with those by univariate analyses, nor by either account. Compared with the extant evidence supporting the pattern difference between objects of expertise (e.g., higher classification accuracy for chess boards in chess experts), the control stimuli were scene pictures (Bilalić, 2016), whereas, in the current study, the control stimuli were everyday objects. Future studies will be needed to evaluate better the effect of control conditions in affecting MVPA outcomes. In addition, while there are studies that combined both multivariate and univariate analyses (Bilalić, 2016), other studies have highlighted the importance of “targeted tests of the informational content and/or dimensionality of activation patterns,” as they are “critical for drawing strong conclusions about the representational codes that are indicated by significant MVPA results” (Farah, 2000). In the current study, the FFA activation differences, as shown in Figures 6, 10, seem to be consistent with the MVPA searchlight results in the 11th neurovault map while also cannot exclude the before training variabilities among trainees in either group (e.g., the 13th and 14th neurovault maps). In short, while univariate analysis results are more supportive of the experience modulation account than those provided by multivariate analyses, it may not be easily attributable to any or a few dimensions that are (or are not) discoverable or impacted by Greeble training. Future analysis should empirically evaluate more testable hypotheses explaining the different results yielded by univariate and multivariate analyses.

The RT differences near the end of training between the Gauthier97 and Gauthier98 paradigms were ~500 and 1,000 ms, respectively. As depicted in Figure 11, participants in the Gauthier98 training may have undergone more processing complications, which rendered the decision times of judging whether the image matched the label proportionally longer. The more uncertainty during the decision processes (or more proposed steps), the longer (800 and 1,200 ms for the named and unnamed Greeble versions in Gauthier98) the average reaction times at the end of training. These putative processes not only help explain the observed reaction time (RT) differences among different conditions but also underlie why Gauthier97 may be a relatively appropriate paradigm for the fMRI effect of Greeble training.

At the outset of this Greeble training study, the authors struggled with the choice of the symmetry (e.g., symmetrical or asymmetric) of Greeble stimuli versions. Our final choice of using asymmetric Greebles was based on the following concern: the observed effects of FFA activities on Greebles, faces, or objects, if found as predicted, might still be attributed to the adage of “Greebles look like faces” (Farah, 2000), even though Greebles are the same face-like to both experts and novices (but FFA only respond significantly to the former after expertise acquisition). If a set of totally non-face-like stimuli, such as the symmetric Greebles of the present study, could be trained to drive FFA significantly after extensive training to enable automatic processing at the subordinate level, then the face likeness may be less of an issue. Despite this, it may still be argued that the current study has not replicated the results of Gauthier et al. (1999) and Brants et al. (2011) because of the different Greeble versions. Our replies are as follows: (a) our Gauthier98 behavioral results were similarly comparable to those in Brants et al. (2011), providing evidence that Brants et al. may have adopted this sub-optimal training paradigm, different from those in Gauthier et al. (1999) and the Gauthier97 of the current study. The similar behavioral response patterns [c.f. Figures 3A, C to figure 3 of Gauthier97, as well as the Figures 3B, D to figure 4 of Gauthier98, and Brants et al. (2011), Figure 3], the dissimilarity among behavioral results from each paradigm, plus that the lack of literature support for the effect of stimulus asymmetry in short-term training provide converging evidence against the existence of stimulus symmetry in affecting short-term training; (b) in terms of featural selectivity, FFA has been shown to prefer stimulus symmetry (Caldara et al., 2006), properties strongly associated with faces (Caldara and Seghier, 2009), but the effect (or trainability) of symmetry is yet to be quantified, thereby the effect of stimulus symmetry onto the training effect awaits further assessment; and (c) currently there have been four studies comparing the training effects of both symmetric (Gauthier et al., 1999; Brants et al., 2011) and asymmetric Greebles (Kung et al., 2007 and the current study), while the effect of task (e.g., 1-back identity vs. passive viewing) has been shown to affect the observed training effect in the FFA (i.e., the 1-back task, due to its increased task demand, rendered the before-training FFA activities for Greebles relatively higher, and therefore obscured the later space of increase for Greebles activities with training). Whether such a cross-symmetry comparison could be made is still one of the potential research questions for future study.

The current study, though targeted at one specific article (Brants et al., 2011), was among the literature discussing the functional role of FFA, including three well-known positions (throughout 1997–2010): face specificity, perceptual expertise, and face network approach. Indeed, the first two positions have been exchanged for decades about their relative merits, while the 3rd position, the face network approach, also received extensive support but with less heated debates. For example, the famous Haxby's (Haxby et al., 2001) study initiated the 1st definition of multi-voxel pattern analysis (MVPA), namely the correlations between patterns of multiple voxels within the chosen ROI(s), functionally or anatomically defined. This approach, now contained in packages like CoSMoMVPA, sidelines reviewer 1's point that in the early stage of the fMRI development, the univariate GLM contrasts and then the subject-specific ROIs do have limitations (e.g., univariate only) and biases (e.g., Friston's critique of functional localizers, Friston et al., 2006), but it was precisely these methodologies that made the heated exchanges between the face specificity and perceptual expertise positions possible—without such procedural adherence, the comparison would be between apples (Kanwisher vs. Gauthier/Tarr, etc.) and oranges (Haxby) because they are not comparable on the same methodological background. On the one hand, we (the authors) would always love, and are kept amazed by, the methodological innovations that shed new light on the fMRI field: MVPA, hyperalignment (Haxby et al., 2020), and DNN/CNN (O'Toole and Castillo, 2021), to name just a few recent breakthroughs. These innovations hardly share a common focus. Rather, they diverge to enlighten us with the multifaceted complexities of the human brain. On the other hand, the previous exchanges between perceptual expertise and face specificity, although fruitless to some, sharpen researchers' minds on how the purported analysis methods (e.g., ROI overlap comparison, NIE, ROI-behavior correlations), with their background assumption checks, fare on any given proposal. These latter practices still have philosophical and reasoning merits, despite their 20 years of age (being “90s”). Both are quintessential learning ingredients for cognitive neuroscientists.

Concerning future research opportunities, one of the possible extensions could be to incorporate the lasting effects of training into the design. One general observation for Greeble experts, soon after their last after-training fMRI scan, was how soon they quickly forgot the associated Greeble shapes and names (though there were exceptions). Future training experiments, if adopted, could further investigate the extinction effects and how soon they could be quickly recovered (and their associated brain mechanisms). Additional fMRI analysis methods, such as functional connectivities (O'Reilly et al., 2012), representational similarity analysis (Kriegeskorte et al., 2008), or even deep neural network approaches, could all be viable options. Finally, the effect of FFA adaptation, except its usefulness as a companion index of expertise acquisition, could also be tested in natural experts (such as bird or car experts), further extending its reliability and validity as an alternate, or even the primary, index of perceptual experience.

## Data availability statement

The datasets presented in this study can be found in online repositories. The names of the repository/repositories and accession number(s) can be found in the article/supplementary material.

## Ethics statement

The studies involving humans were approved by NCKU Research Ethics Committee. The studies were conducted in accordance with the local legislation and institutional requirements. The participants provided their written informed consent to participate in this study.

## Author contributions

C-CK conceived the experiment. C-YC developed the code. KL carried out the experiments. KL, C-YC, HJ, and L-SW analyzed the data. KL, HJ, and L-SW prepared the figures. KL, L-SW, and C-CK discussed, revised, and completed the draft. All authors reviewed and approved the final manuscript.
