# Supplementary material for: Is increased activation in the fusiform face area to Greebles a result of appropriate expertise training or caused by Greebles' face likeness?
Source: Front Neurosci. 2023 Oct 17;17:1224721. doi: 10.3389/fnins.2023.1224721 (PMC10616304; doi:10.3389/fnins.2023.1224721)
Supplement: Supplementary file 1 [file Data_Sheet_1.PDF]

Supplementary data analysis

1. Nonlinear regression fitting of the reaction times of the verification task across each training session of both Gauthier97 and Gauthier98 paradigms

The response time decreased as the training session increased and gradually entered a plateau period. Based on these features, we further explored the plateau difference (β_1) between Gauthier97 and Gauthier98 by adopting the exponential decay nonlinear regression model.

$$y=\beta_1+\beta_2 e^{(\beta_3 x)}$$

The results showed that the plateaus of Greeble Gender's response time (β_1=745.04, SDE = 119.16, R^2=0.34) and Greeble Individual's response time (β_1=960.39 SDE=354.31, R^2=0.23) in Gauthier98 group were significantly different. However, the response time plateau of the Greeble Gender (β_1=619.83，SDE= 97.75，R^2=0.59), Greeble Individual (β_1=390.87，SDE=308.22；R^2=0.59), and Greeble family (β_1=617.75，SDE=184.07，R^2=0.50) in Gauthier97 group were very close. Therefore, the results of t-test and nonlinear regression both show that the training effect of Gauthier97 is significantly better (e.g., larger R^2) than Gauthier98.

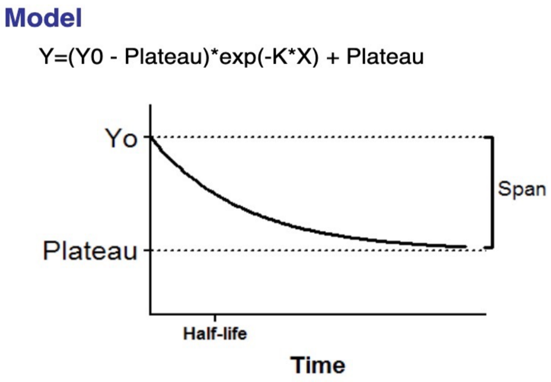

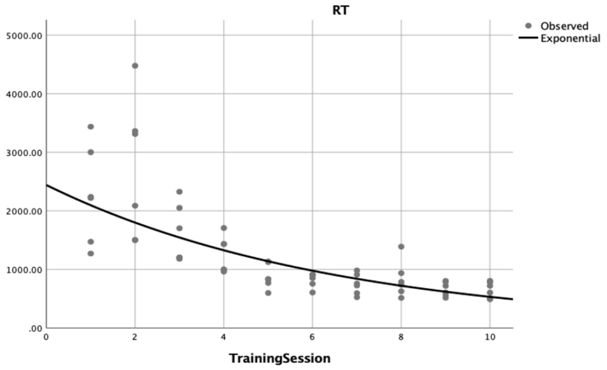

Gauthier97	estimate	SDE	LB	UB	R^2
Gender_plateau	619.832018	97.746168	422.96113	816.702906	0.591
Individual_plateau	390.867986	308.216874	-226.325659	1008.061631	0.587
Family_plateau	617.747606	184.065656	249.162487	986.332725	0.503

Gauthier98	estimate	SDE	LB	UB	R^2

Gender_plateau	745.037669	119.162866	506.418146	983.657192	0.327
Individual_plateau	960.387361	354.310994	250.891834	1669.882889	0.23

2. About the statistical tests of the training effect (behavioral data analysis). As it turns out, when developing the Psychtoolbox code for Gauthier97 and Gauthier98 training paradigms (also archived in osf.io folder), the individual RT for each trial was not saved for both naming and verification tasks of the Gauthier97 paradigm, but saved for both named and unnamed Greeble trails in Gauthier98. This is the primary reason why in Figure 4 (for Gauthier97) of the main text, the correct RT plots for each condition (gender, family, and individual) of each individual do not contain error bars, but do contain those in Figure 5 (of individual RTs in Gauthier98). Therefore, in the ANOVA analyses of Gauthier97 training RT results, only the final average of each of the 8 subjects was taken into the final N, rendering the ANOVA results relatively noisy. Session-wise ANOVA revealed that only in session 2 were the ANOVA results significantly different among the 3 levels (gender, family, and individual).

As for reviewer 2's suggestion of running multiple corrections for comparisons over multiple training sessions, we do not think it necessary because (a) although our question was to ask in which session did the family and individual RT began to converge (or statistically insignificant), the answer could not be any of the 10 training sessions: because other constraints, such as the initial RTs among the 3 levels have to be big (and significantly different), and then gradually reduced to the point of convergence. Therefore, the question has 3 levels of constraints to make the final answer either session 5 (statistically) or 7 (by eyeballing, see Fig. 3). Therefore, we do have reasons for not using corrections over multiple comparisons. The "mean response times in the individual level were longer than those in the family, and the gender levels [the 1st ~5th session: $F(2,21)= 3.55$, $p = 0.047$, $\eta2 = 0.253$] (reported in pp. 16 of the article) was done by combining the first 5 training sessions of Gauthier97.

All the above-mentioned 2 analysis scripts (using SPSS) are archived in https://osf.io/5tm9v/, under \Behavioral_analysis\supplemental_analyses.
